# Frequency and Predictors of Courses Repetition, Probation, and Delayed Graduation in Kashan Faculty of Nursing and Midwifery

**DOI:** 10.5812/nms.9920

**Published:** 2013-12-10

**Authors:** Zahra Tagharrobi, Negin Masoudi Alavi, Esmail Fakharian, Fakhrossadat Mirhoseini, Sayyed Asghar Rasoulinejad, Hossein Akbari, Hossein Ameli

**Affiliations:** 1 Department of Health and Management Nursing, Kashan University of Medical Sciences, Kashan, IR Iran; 2 Trauma Nursing Research Center, Kashan University of Medical Sciences, Kashan, IR Iran; 3 Department of Neurosurgery, Kashan University of Medical Sciences, Kashan, IR Iran; 4 Department of Anesthesiology, Kashan University of Medical Sciences, Kashan, IR Iran; 5 Department of Statistics and Public Health, Kashan University of Medical Sciences, Kashan, IR Iran; 6 Postgraduate Studies Office, Kashan University of Medical Sciences, Kashan, IR Iran

**Keywords:** Educational Measurement, Educational Status, Student Dropouts, Education, Medical

## Abstract

**Background:**

Course failing and delayed graduation are important concerns in educational systems. The reasons of these educational failures need to be clarified.

**Objectives:**

This study was designed to determine the academic failure rate and its predictors in Nursing and Midwifery Students in Kashan University of Medical Sciences.

**Materials and Methods:**

In this cross-sectional study, the records of all the students graduated in Nursing and Midwifery faculty during 18 years (1986 - 2003) were evaluated (1174 graduates). The demographic variables and the educational situation were recorded. The frequency of course repetition, probation, and delayed graduation were determined and the data were analyzed using the chi-square and logistic regression tests.

**Results:**

The frequency of course repetition, probation, and delayed graduation was reported to be 19.25%, 3.9% and 19.85%, respectively. Gaining Low grade in high school, transferring from other universities, having special quota, and transferring temporarily to other universities were mentioned as the risk factors of academic failure. The major had a significant relationship with academic failure. Day time students had more course failure and night time students stayed longer in the university.

**Conclusions:**

The individual characteristics, educational background and admission criteria had showed relation with academic failure. Vulnerable students should be identified and educational supports should be provided for these students.

## 1. Background

The quality of education is an important concern in the educational systems (1). On the other hand, evaluating the efficiency of educational systems is an important component of quality improvement (2). One of the most important aspects of efficiency evaluation in educational systems is the evaluation of academic failure and its familial and psychosocial outcomes for the students (3-5). Academic failure has been widely defined worldwide; however, the common part of all the definitions is that academic failure refers to the inability to finish the formal course of education successfully (6). There are different criteria for evaluation of academic failure (2) including course repetition, academic probation (hereinafter briefly referred to as probation), and delayed graduation. Academic failure is a prevalent educational issue in academic settings (7). The most common criteria for evaluating academic failure, in studies conducted in our country, were probation and students’ overall average (4-6, 8-10). The results of these studies vary widely because of the difference in the study settings and data collection tools. Several researchers have addressed the difference in the rate of academic failure in different fields of studies and have considered different reasons for this difference (5). Raoufi (2008) reported that 10.6% of nursing students affiliated to Tabriz University of Medical Sciences, Tabriz, Iran, have experienced academic failure (4). On the other hand, the rate of academic failure among nursing students affiliated to AJA University of Medical Sciences, Tehran, Iran, has been 16.9% (9). Hazavehei reported that 4.81% of nursing students affiliated to Hamedan University of Medical Sciences, Hamedan, Iran, have experienced probation at least once. He also reported that the minimum rate of course repetition in two subsequent semesters was 13% (1). Edalatkhah also estimated that the prevalence of probation among midwifery and operating room students affiliated to Ardebil University of Medical Sciences, Ardebil, Iran, was 4.1% and 4.7%, respectively (5). Most researchers believed that an important intervention for preventing academic failure is the identification of at-risk students and related risk factors (1, 6). Many factors affect students’ academic failure. They have been classified as personal and familial, socioeconomic, and educational factors (11). The effects of factors such as university admission strategies (12-17), high school average, educational background (18, 19), entrance exam scores (20), age (21), gender (22-24), delayed university entrance (25), marital status (2), employment (26), and familial, cultural, and socioeconomic background (25) have been investigated in previous studies. Most of these studies have been conducted exclusively on nursing students (2, 24, 26-30). There is still considerable controversy in terms of the effects of the aforementioned factors on students’ academic failure (27, 31-37). On the other hand, beside the sample size, sampling method, study instrument, and employed statistical tests may be related to the study population and setting (5). The factors affecting the educational failure need to be studied in different settings and places to explore the different dimensions of this negative educational outcome.

## 2. Objectives

The first aim of this study was to determine the academic failure rate - as measured by indices such as course repetition, probation, and delayed graduation rate - in nursing graduate students affiliated to Kashan Faculty of Nursing and Midwifery, Kashan, Iran. The second aim of the study was to identify the factors affecting academic failure. 

## 3. Materials and Methods

This was a cross-sectional descriptive study conducted in Kashan University of medical sciences. The educational records of all the students who had entered the Kashan Faculty of Nursing and Midwifery between 1986 and 2003 were retrieved and included in the study. Guest students and students moved from Kashan University to other universities were excluded from the study. The students transferred to this faculty were included in this study. Totally, the educational records of 1174 students were retrieved from the university archives and included in the final analysis. The study questionnaire included questions about students’ age, gender, marriage, residency, field of study in high school, overall diploma and pre-university course average, rank in the National University Entrance Exam, university entrance exam quota, the interval between graduation from high school and entrance to the university, academic field and degree of study, and academic failure indices - including course repetition (name of the course and number of repetition), probation, and delayed graduation. Moreover, for the students studying in two-year programs, data regarding the overall average of these programs and the interval between graduation from these programs and entrance to the next two-year programs was also collected. In the course repetition variable, any courses repeated twice or more by a single student were recoded as one time. Delayed graduation in two-year and four-year programs was defined as more than four and eight semesters of education. Moreover, for calculating the mean of academic education length, the records of dismissed students and students dropping out of university were excluded. On the other hand, as the number of missed values in the variables of “rank in the National University Entrance Exam” and “the overall average in the two-year program” was high, they were excluded from the final analysis. Study data was collected by educational staff working in the university archives. Faculty members and educational staff affiliated to our university checked and confirmed the content validity of the questionnaire. 

### 3.1. Data Analysis

The Statistical Package for Social Sciences, SPSS 16, was used for data management and analysis. Initially, the frequency of course repetition, probation, and delayed graduation was calculated. Thereafter, the Chi-square test, Fisher’s exact test, and binary logistic regression model were employed to identify probable correlations between the intended factors and academic failure. In the next step, to remove the intervening effects of the factors, we entered all the intended factors in the multinomial logistic regression model. A recent model, we employed the Enter method. The level of significance was considered less than 0.05. 

### 3.2. Ethical Considerations

Helsinki agreement content was considered in the design of this study design. Our university-affiliated Institutional Review Board and Ethics Committee approved the study. We coded the questionnaires using numbers to maintain the confidentiality of the students’ data. 

## 4. Results

Study findings showed that 803 students (68.4%) were female, 112 students (9.5%) were married, 1102 students (93.9%) had diplomas in biological sciences, and 966 students (82.2%) had used Regions quota in the National University Entrance Exam. Considering the field of study, 285 (24.3%), 230 (19.6%), and 659 (56.1%) of the study subjects were midwifery, operating room, and nursing students, respectively. Four hundred and four students (34.4%) were originally from Kashan. Considering the academic degree, 381 students (32.5%) were studying in two-year programs, 625 students (53.2%) in continuous baccalaureate programs, and 168 students (14.3%) in discrete baccalaureate programs. Eight hundred and nine students (68.9%) were studying on day time courses. The mean of students’ overall diploma average was 14.86 ± 2.7. On the other hand, the mean of midwifery, operating room, and nursing students’ overall diploma average was 15.56 ± 2.1, 14.93 ± 2.32, and 14.53 ± 2.26, respectively. The mean of interval between graduation from high school and entrance to the university was 1.44 ± 1.7 years and the mean of students’ age while entering the university was 20.69 ± 3.37. The midwifery, operating room, and nursing students’ overall average in the university was 16.09 ± 1.1, 15.4 ± 1.2, and 15.68 ± 1.25, respectively. Moreover, the mean of academic education length for two- and four-year programs was 4.27 ± 0.57 and 8.27 ± 0.61 semesters, respectively. Totally, 46 students (3.9%) had a history of probation; accordingly, 26 (2.2%), 11 (0.9%), eight (0.7%), and one students (0.1%) had a history of probation for one, two, three, and four semesters. Uni- and multi-variate analysis revealed that factors such as residency, field of study in high school, marital status, academic field of study, studying in day time and night time, history of inter-university exchange, the interval between graduation from high school and entrance to the university, and the interval between graduation from the two-year programs and entrance to the next two-year program have no significant effects on probation incidence. Table 1 illustrates the risk factors for probation. 

**Table 1.** The Frequency Distribution of the Assessed Samples Based on Probation Status and Its Risk Factors

Probation Risk Factors	Negative	Positive	Test Type and Result			
			Univariate		Multi- Variate	
			P value	Crude Odd Ratio	P value	Adjusted Odd Ratio
**Gender, No. (%)**			< 0.0001		0.98	
Female	783 (69.4)	20 (43.5)		1		-
Male	345 (30.6)	26 (56.5)		2.95 (1.62 - 5.25)		-
**Quota type, No. (%)**			< 0.0001		0.004	
Regions	942 (85.8)	24 (53.3)		1		1
Others	156 (14.2)	21 (46.7)		5.28 (2.87 - 9.71)		3.49 (1.51 - 8.08)
**Courses type, No. (%)**			0.16		0.22	
Night	355(31.5)	10(21.7)	-			-
Daily	773(68.5)	36(87.3)	-			-
**Temporary transferring to other universities, No. (%)**			0.02		0.02	
No	1104 (97.9)	42 (91.3)		1		1
Yes	24 (2.1)	4 (8.7)		4.38 (1.46 - 13.16)		5.19 (1.36 - 19.88)
**High school grade point average, Mean (SD)**	14.94 (2.24)	12.92 (2.25)	< 0.0001	0.67 (0.57 - 0.77)	0.002	0.74(0.61 - 0.89)
**Age when entering university, Mean (SD), y**	20.65 (3.34)	21.70 (3.77)	0.04	1.07 (1.003 - 1.15)	0.13	-

Study findings showed that 226 students (19.25%) had experienced course repetition for one (124 students; 10.6%), two (48 students; 4.1%), three (29 students; 2.5%), or four and more (25 students; 2.1%) courses. The most-frequently repeated courses in the nursing field of study were English language (43.4 per 1000 students), Physiology (33.9 per 1000 students), and Biochemistry (25.8 per 1000 students), respectively. In the midwifery field of study, the most-frequently repeated courses were Biochemistry and Physiology (28.1 per 1000 students), Obstetrics1 (21.1 per 1000 students), and English language, Medical-Surgical nursing, and Applied Laboratory Tests (10.5 per 1000 students), respectively. On the other hand, the most-frequently repeated courses in the operating room field of study were Biochemistry (108.7 per 1000 students), Pathology (52.2 per 1000 students), and Physiology (43.5 per 1000 students), respectively. The results of uni- and multi-variate analyses showed that factors such as residency, field of study in high school, marital status, history of inter-university exchange, the interval between graduation from high school and entrance to the university, the interval between graduation from the two-year programs and entrance to the next two-year programs, and the students’ age while entering university had no significant effects on the incidence of course repetition. Table 2 illustrates the risk factors for course repetition. 

**Table 2.** The Frequency Distribution of the Assessed Samples Based on Course Repetition Status and its Risk Factors

Course Repetition Risk Factors	Negative	Positive	Test Type and Result			
			Univariate		Multi- Variate	
			P value	Crude Odd Ratio	P value	Adjusted Odd Ratio
**Gender, No. **(%)			< 0.0001		0.31	
Female	671 (70.8)	132 (58.4)		1		-
Male	277 (29.2)	94 (41.6)		1.73 (1.28 - 2.33)		-
**Quota type, No. (%)**			< 0.0001		< 0.0001	
Regions	801 (87.2)	165 (73.7)		1		1
Others	118 (12.8)	59 (26.6)		2.43 (1.7 - 3.46)		2.49 (1.52 - 4.07)
**Major, No. (%)**			< 0.0001		< 0.0001	
Nursing	526 (55.5)	133 (58.8)		1		1
Midwifery	256 (27)	29 (12.8)		0.45 (0.29 - 0.89)		1.03 (0.49 - 2.15)^a^
Operation Room	166 (17.5)	64 (28.3)		1.53 (1.08 - 2.15)		4.91 (1.77 - 13.62)
**Courses type, No. (%)**			0.006		0.02	
Night	312 (32.9)	53 (23.5)		1		1
Daily	636 (67.1)	173 (76.5)		1.6 (1.14 - 2.24)		1.8 (1.10 - 2.93)
**Temporary transferring to other universities, No. (%)**			0.03		0.08	
No	930 (68.1)	216 (95.6)		1		-
Yes	18 (1.9)	10 (4.4)		2.39 (1.09 - 5.26)		-
**Study Degree, No. (%)**			< 0.0001		0.22	
Two-year program	301 (31.8)	80 (35.4)		3.17 (1.71 - 5.88)		-
Continuous baccalaureate	492 (51.9)	133 (58.8)		3.22 (1.77 - 5.86)		-
Discrete baccalaureate	155 (16.4)	13 (5.8)		1		-
**High school grade point average, mean (SD)**	15.16 (2.22)	14.04 (2.19)	< 0.0001	0.83 (0.77 - 0.88)	0.001	0.86 (0.78 - 0.94)
**Pre-university grade average mean (SD)**	17.10 (1.31)	16.10 (1.76)	0.02	0.64 (0.44 - 0.93)	It was not entered in the model	

^a^ Non-significant

The study findings revealed that 233 students (19.85%) - 120 (10.22%) in the two-year and 113 (9.63%) in the four-year programs - had experienced delayed graduation. Accordingly, in the four-year programs, 77 (12.32%), 31 (4.96%), and 5 students (0.8%) had experienced one, two, and three or more semesters of delayed graduation, respectively. On the other hand, the length of delayed graduation in the two-year programs was either one (106 students; 19.31%) or two and more (14 students; 2.55%) semesters. The results of uni- and multi-variate analyses showed that factors such as field of study in high school, the interval between graduation from high school and entrance to the university, the interval between graduation from the two-year programs and entrance to the next two-year program, and overall average of the one-year pre-university course had no significant effects on the incidence of delayed graduation. Table 3 illustrates the risk factors for delayed graduation. 

**Table 3.** The Frequency Distribution of the Assessed Samples Based on Delayed Graduation and Its Risk Factors

Delayed Graduation Risk Factors	Negative	Positive	Test Type and Result			
			Univariate		Multi- Variate	
			P value	Crude Odd Ratio	P value	Adjusted Odd Ratio
**Gender, No.(%)**			0.009		0.92	
Female	605 (73.2)	150 (64.4)		1		-
Male	222 (26.8)	83 (35.6)		1.51 (1.11-2.05)		-
**Place of residence, No. (%)**			< 0.0001		0.97	
Kashan	537 (66.2)	115 (50.2)		1		-
Others	274 (33.8)	114 (49.8)		1.94 (1.44 - 2.62)		-
**Marital status, No. (%)**			< 0.0001		0.2	
Single	756 (93.1)	184 (79.9)		1		-
Married	56 (6.9)	47 (20.3)		3.45 (2.27 - 5.24)		-
**Quota type, No. (%)**			< 0.0001		0.02	
States	729 (89.2)	163 (71.5)		1		1
Others	88 (10.8)	65 (28.5)		3.3 (2.2 - 4.75)		2.19 (1.13 - 4.23)
**Major, No. (%)**			< 0.0001		0.02	
Nursing	397 (48)	170 (73)		1		1
Midwifery	233 (28.2)	39 (16.7)		0.39 (0.27 - 0.57)		1.85 (0.72 - 4.74)
Operation Room	197 (23.8)	24 (10.3)		0.29 (0.18 - 0.45)		0.37 (0.09 - 1.49)
**Courses type, No. (%)**			< 0.0001		< 0.0001	
Daily	640 (77.4)	76 (32.6)		1		1
Night	187 (22.6)	157 (67.4)		7.07 (5.14 - 9.72)		21.81 (11.44 - 41.60)
**Temporary transferring to other universities, No. (%)**			0.02		0.001	
No	815 (98.5)	224 (96.1)		1		1
Yes	12 (1.5)	13 (5.6)		2.73 (1.14 - 6.56)		6.23 (2.05 - 18.96)
**Transferring from other universities, No. (%)**			0.03		< 0.0001	
No	805 (97.3)	220 (94.4)		1		1
Yes	22 (2.7)	13 (5.6)		2.16 (1.07 - 4.36)		5.55 (2.25 - 13.66)
**Study Degree, No. (%)**			< 0.0001		0.11	
Two-year program	330 (39.43)	32 (13.73)		0.09 (0.05-0.14)		-
Continuous baccalaureate	421 (50.91)	113 (48.5)		0.23 (0.16-0.34)		-
Discrete baccalaureate	76 (9.19)	88 (37.77)		1		-
**High school grade point average, Mean (SD)**	15.16 (2.2)	14.04 (2.19)	< 0.0001	0.80 (0.75 - 0.86)	< 0.0001	0.80 (0.71 - 0.90)
**Age when entering university , Mean (SD), y**	20.2 (2.8)	22.4 (4.3)	< 0.0001	1.19 (1.14 - 1.24)	0.34	-

## 5. Discussion

Study findings showed that special quotas and low diploma average significantly affect course repetition, probation, and delayed graduation. Moreover, we found that a history of being a guest student in other universities increases the risk for probation and delayed graduation. Findings also showed that studying in day time courses is a risk factor for course repetition while studying in night time courses and inter-university exchange are risk factors for delayed graduation. Finally, we found that the academic field of study has a significant effect on course repetition and delayed graduation. We found that 3.9% of students had experienced probation. The prevalence of probation in Kashan Faculty of Allied Health Sciences (5), Ardebil University of Medical Sciences (2), and Tabriz University of Medical Sciences (4) has been 9.2%, 7.4%, and 4.4%, respectively. The main reasons for such differences in the probation rate in different settings may be the difference in study population and interval in different studies and the difference in teaching strategies and evaluation criteria of different educational systems. Our findings also revealed that 19.25% of students had experienced course repetition. Other studies have reported that the prevalence of course repetition in dentistry (38), medical (33), and allied health sciences students (2) has been 36.73%, 16.72%, and 28.7% respectively. Hazavehei also found that the frequency of repetition of at least one course in two successive semesters is 13% (1). We found that the most-frequently repeated courses were English language, Biochemistry, and Physiology. However, the most-frequently repeated courses in the Hazavehei study were English language and Biostatistics (1). The frequency of delayed graduation in our study was 19.85%; however, the frequency of delayed graduation in Kashan Faculty of Allied Health Sciences (2) and Faculty of Medicine (33) was 14.1% and 32.3%, respectively. In this study, the results of uni-variate analysis showed that the students’ age had no significant effect on academic failure; however, the results of multi-variate analysis revealed that for every year increase in the students’ age in the time of entering university, the risk of probation and delayed graduation increases by 7% and 19%, respectively. Some studies also reported the negative effect of age on academic performance (2, 39). Regarding the reasons for such negative effect of age, scholars believed that older students experience more stressful events during their academic life and hence, have poorer academic performance compared to their younger counterparts (2). On the other hand, some studies have reported a positive effect of age on academic performance. The reason is that older students are more experienced, motivated for job promotion, and supportive networks (2, 36). We also found that low diploma average is a risk factor for academic failure. Other studies also reported the same finding (20, 30, 34). Scholars believed that poor academic performance in students with poor educational background is due to their unfamiliarity with effective learning strategies. Therefore, teaching these strategies to freshmen students may improve their academic performance (6). The results of multi-variate analysis revealed that students’ gender has no significant effect on academic failure. Other studies also reported the same findings (9, 31, 35). However, the results of uni-variate analysis, along with other studies (1, 22), showed that academic failure is more prevalent in male students. the reason of this fact has been related to the male students’ concerns about their unemployment, job prospect, and socioeconomic status (6). Further studies are needed to determine other reasons for this issue. The results of uni-variate analysis revealed that being native of Kashan is a risk factor for delayed graduation; however, the results of multi-variate analysis, in parallel with the results of other studies (1, 35), showed that students’ residency has no significant effect on delayed graduation. Factors such as easier access to educational resources and consultants in university dormitories, more serious educational competition among dormitory students, and native students’ familial responsibilities and concerns may mediate the effect of being a native on delayed graduation. The results of a study (2) revealed that, for using of quota, native students usually enter fields of study that are not interested in. The rational outcome of being uninterested in the field of study is poor academic performance that results in academic failure. The results of multi-variate analysis revealed that marital status is not a risk factor for academic failure. Gheibi (35) and Hazavehei (1) also reported the same finding. However, the results of several studies showed that marital status negatively affects academic performance (3, 6, 8). The results of uni-variate analysis, in our study, showed that the risk for delayed graduation in married students is 3.5 times more than their single counterparts. Scholars believed that negative stressful events associated with marital status (2), inadequate welfare services available to the married students, and their employment in false jobs can affect their academic performance (10). Our findings showed that academic failure was more prevalent in students using particular kinds of university entrance quotas. Some of the previous studies also reported the same finding (5, 8, 32) while other studies reported a contrary finding (34, 35). Our findings showed that the interval between graduation and the two-year programs and entrance to the next two-year programs has no significant effect on students’ academic failure. Bakouei (2010) also conducted a study in Babol University of Medical Sciences and reported the same finding (11). However, other studies have reported a significant correlation between these two variables (3, 8, 35). It is believed that those students who experience a delay in entering higher level education are less competent and more prone to academic failure. We found that a history of being a guest student in other universities increases the risk for probation and delayed graduation. The results of multi-variate analysis also revealed that delayed graduation is more prevalent in students having a history of inter-university exchange. Studies showed that exchange rate is more prevalent among students with poor academic performance (32). Moreover, the difference in the arrangement of courses in different universities is another reason for higher prevalence of delayed graduation in students having a history of inter-university exchange. We also found that studying in day time courses is a risk factor for course repetition while studying in night time courses is a risk factor for delayed graduation. Because of higher part-time employment rate among night time students and the difference between arrangement of courses in day time and night time courses, higher delayed graduation rate among night time course students is inevitable. Moreover, Arulampalam (2004) has confirmed the effect of tuition fees on students’ academic performance (7). The study findings also revealed that the students’ field of study affects the frequency of course repetition and delayed graduation. However, multi-variate analysis showed that the students’ academic degree has no effect on academic failure. Some studies also reported that field and degree of study can affect the students’ academic performance (5). The reasons for the difference in academic failure rate in various fields and degrees of study are probably the difference in their curriculum content and evaluation criteria. It is noteworthy that the difference in time, setting, data collection tools, sampling methods, and statistical analysis methods of different studies may be the possible sources of difference in the frequency and predictors of academic failure in different academic settings. The almost high academic failure rate among midwifery, operating room, and nursing students affiliated to our faculty necessitates university managers to pay more attention. Moreover, the findings highlighted the important role of factors such as fields and course (either day time or night time) of study, overall diploma average, university entrance exam quota, the history of inter-university exchange, and the history of being a guest student in other universities related to academic failure. Therefore, modifying the university admission processes and teaching strategies, improving welfare services and educational facilities, designing more supportive interventions, putting the Educational Consultants Regulation in practice, empowering the students’ supervisors and advisers, and employing competent consultants in faculties of nursing and midwifery may improve students’ academic performance. Accordingly, the following strategies are recommended:

Considering the students overall diploma average as a university entrance exam criteria;
Providing more educational support for students who use some particular kinds of university entrance quotas;
Supervising regularly and follow up of academic performance of guest students;
Including teaching of effective learning strategies in the academic curriculum;
Providing the compensatory programs and educational consultation to the students with poor educational background;
Supervising the students having a history of inter-university exchange; and
Conducting interventional studies in the area of academic failure prevention.

This study was conducted using the former students’ records available in the university archives. Therefore, we could not investigate the effect of other personal and environmental factors on the students’ academic failure rate. Consequently, we recommended other interested researchers to conduct their studies on the students who are currently under education.
